# Effect of gut microbiome-derived metabolites and extracellular vesicles on hepatocyte functions in a gut-liver axis chip

**DOI:** 10.1186/s40580-022-00350-6

**Published:** 2023-01-16

**Authors:** Seong Goo Kang, Yoon Young Choi, Sung Jun Mo, Tae Hyeon Kim, Jang Ho Ha, Dong Ki Hong, Hayera Lee, Soo Dong Park, Jae-Jung Shim, Jung-Lyoul Lee, Bong Geun Chung

**Affiliations:** 1 Department of Biomedical Engineering, Sogang University, Seoul, 04107 Korea; 2 Institute of Integrated Biotechnology, Sogang University, Seoul, 04107 Korea; 3 R&BD Center, hy Co., Ltd., Yongin-Si, Korea; 4 Department of Mechanical Engineering, Sogang University, Seoul, 04107 Korea

**Keywords:** Gut-liver axis chip, Metabolites, Microbiome, Exosome, Liver function

## Abstract

Metabolism, is a complex process involving the gut and the liver tissue, is difficult to be reproduced in vitro with conventional single cell culture systems. To tackle this challenge, we developed a gut-liver-axis chip consisting of the gut epithelial cell chamber and three-dimensional (3D) uniform-sized liver spheroid chamber. Two cell culture chamber compartments were separated with a porous membrane to prevent microorganisms from passing through the chamber. When the hepG2 spheroids cultured with microbiota-derived metabolites, we observed the changes in the physiological function of hepG2 spheroids, showing that the albumin and urea secretion activity of liver spheroids was significantly enhanced. Additionally, the functional validation of hepG2 spheroids treated with microbiota-derived exosome was evaluated that the treatment of the microbiota-derived exosome significantly enhanced albumin and urea in hepG2 spheroids in a gut-liver axis chip. Therefore, this gut-liver axis chip could be a potentially powerful co-culture platform to study the interaction of microbiota and host cells.

**Graphical Abstract:**

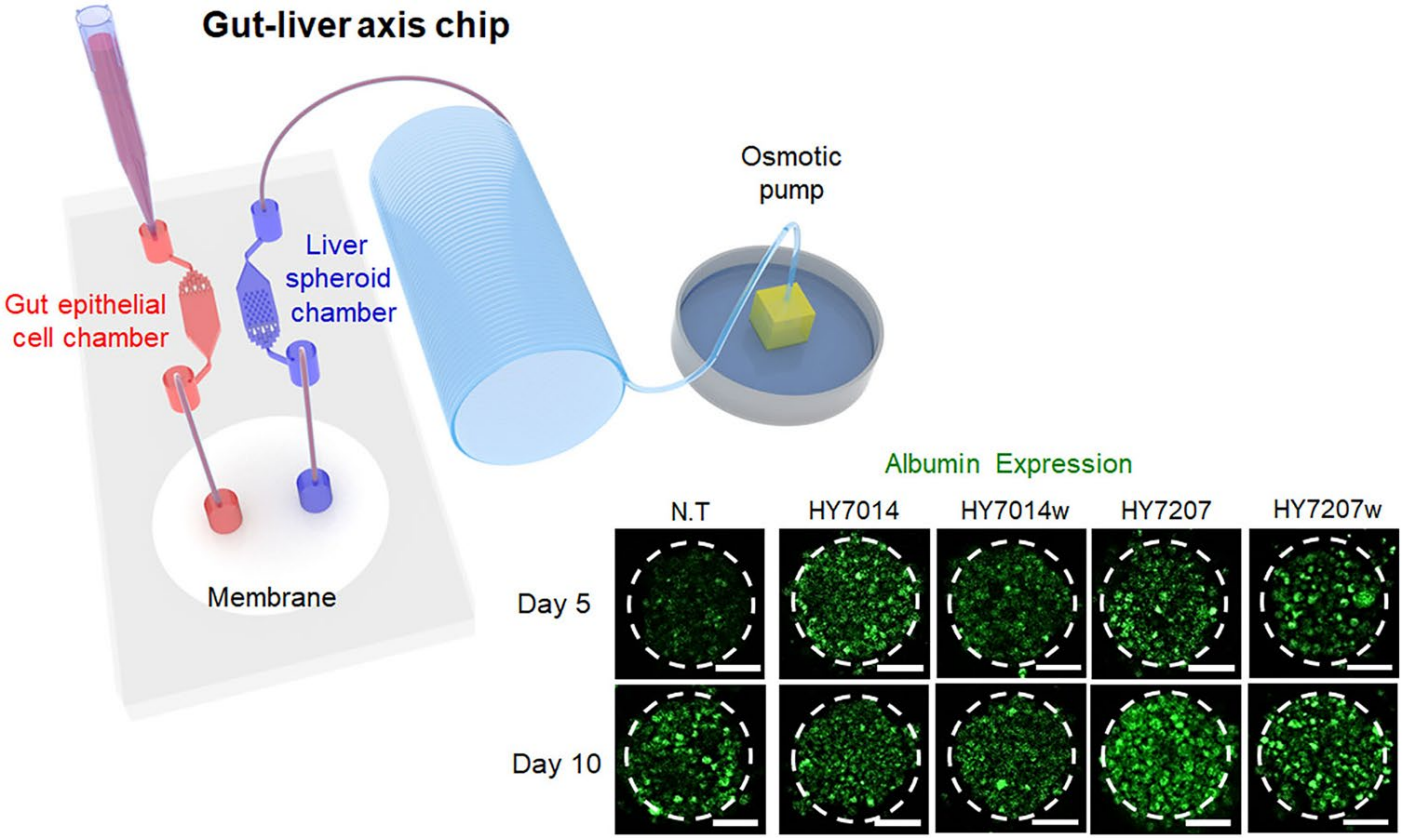

**Supplementary Information:**

The online version contains supplementary material available at 10.1186/s40580-022-00350-6.

## Introduction

The gut-liver axis is a physiologically connected system that enables the control of processing of the gut-derived products, regulation of the metabolic homeostasis, and stability of the immune function. In the last few years, in vitro culture models of the gastrointestinal (GI) tract and liver have independently been established [1, 2]. However, the single organ system only focused on the reconstruction of a specific organ, causing inability of mimicking the complex intercellular signal communications*.* Additionally, current in vitro cell‐based model systems are not able to recapitulate the microenvironment of in vivo tissues. To overcome these limitations, a number of researchers have recently turned their attention to the organ-on-a-chip system for study of the interaction between multi-organs [3, 4]. The organ-on-a chip system aims to development of an advanced in vitro model by mimicking the native tissue environments. Another major beneficial effect of an organ‐on‐a‐chip system is to realize the interaction between different organs [5]. In recent years, the gut-liver-on-chip was integrated to imitate the process of the absorption and metabolic reactions [6, 7]. Most of these studies have focused on the inflammatory reaction of the specific disease based on certain signal pathways. Although these previous studies were applied to recapitulate the disease, the effect of gut microbiome-derived metabolites and extracellular vesicles (EVs) have rarely been investigated. To date, only a few studies have investigated an organ-on-a-chip to explore the microbiome-derived metabolites and EVs [8–10]. Therefore, there is a need to understand how such metabolites or EVs can affect cellular functions at in vivo-like microenvironments.

It is estimated that the gut microbiota contains about 10^15^ microbial cells and more than 22 million microbial genes [11, 12]. With these genes, the gut microbiota can synthesize a number of enzymes from exogenous dietary substrates or endogenous host compounds. [13] As a result, the gut microbiota can produce various metabolites with wide spectrum of bioactivities. These microbial metabolites are crucial actors in a host-microbiota cross-talk. Furthermore, these molecules can act on multiple organs or tissues [14]. According to the advanced metabolomics analysis (e.g., mass spectrometry), a large groups of gut microbiota metabolites have been identified, such as short-chain fatty acids (SCFAs), bile acids, and choline metabolites [15]. These metabolites can induce a series of physiological and pathological functions on hosts and other bacteria, such as modulation of energy metabolism, nutrition absorption, and regulation of gut microbiota composition. The recent evidence of microbe-derived soluble factors also suggests that EVs containing various metabolites can contribute to interactions with other members of the microbial community [16, 17]. Nano-sized EVs represent the novel secretion systems, allowing for spreading of membrane-encapsulated cellular materials (e.g., proteins, nucleic acids, and metabolites) into the extracellular environment [18]. It has been known that EVs can significantly affect the neighboring cells near the secreting cells. EVs not only trigger downstream signals but also transfer genetic materials to the target cells, thereby exerting anti-inflammatory, antiapoptotic, and immunosuppressive effects to promote tissue repairs [19]. Additionally, an EV act as a vitally important intercellular delivery carrier which transfers extensive types of the signals to neighboring cells [20].

The intestine and liver were closely connected by the portal vein and the transports of gut-derived productions interact with the small intestine [21]. The gut-derived productions were also transported across the gut epithelium and absorbed into the bloodstream before reaching the liver by the portal vein [22]. Therefore, the investigation of the gut microbiome-derived products on liver functions in an organ-on-a-chip system is required to understand the effect of gut microbiome-derived products on hepatocytes. Here, we report the development of the gut-liver axis chip system for investigating the effect of gut microbiota-derived metabolites and EVs on hepatocyte functions. A gut-liver axis chip system consisted of a cascade design in which the culture medium could move from the gut and microbiota culture chamber to the three-dimensional (3D) uniform-sized hepG2 spheroid chamber within microwell arrays. A two-chamber separated by a cellulose membrane could allow for culturing gut-microbiota and uniform-sized hepG2 spheroids simultaneously. Therefore, hepG2 spheroids were only affected by molecules derived from the gut microbiota due to the structure of the membrane-separated chamber compartment. We also investigated the effect of EVs on uniform-sized hepG2 spheroid functions using albumin and urea analysis. Therefore, our gut-live axis chip system could be extensively used not only in well-organized liver spheroid formation, but also in the better understanding of microbiota–host cell interaction effect at in vivo-like microenvironments.

## Materials and methods

### Fabrication of gut-liver axis chip and experimental set-up

The gut-liver axis chip was designed using Autocad (Autodesk, CA, USA). The embedded gut and liver on-a-chip master mold was made by two-step lithography process as previously reported [23, 24]. First, SU-8 100 photoresist (MicroChem Corp., MA, USA) was deposited and spin-coated with 2000 rpm for 30 s on a 4-inch silicon wafer and baked at 65 °C for 20 min, 95 °C for 1 h, respectively. Ultraviolet (UV) light was exposed for 40 s with UV aligner (MDA-400LJ, Midas System Co. Ltd, Daejeon, Korea) through a photomask and developed unexposed photoresist for 12 min to fabricated the microchannel. SU-8 100 photoresist was secondly deposited on the patterned wafer, spin-coated at 1000 rpm for 30 s, exposed to UV light for 60 s, and developed to fabricate the microwell array. The polydimethylsiloxane (PDMS)-based gut-liver axis chip mold was prepared using a 10:1 mixture of a silicone elastomer and curing agent (Sylgard 184, Dow Corning Corp., MI, USA). The elastomer mixture was placed in a vacuum desiccator (Lab Companion, Daejeon, Korea) for 30 min to remove air bubbles and polymerized at 80 °C for 1 h. The polymerized gut-liver axis chip molds were treated with in a plasma cleaner (Femto Science, Korea) to bond each other. For an osmotic pump, PDMS cubic chambers (1 × 1 × 1 cm) with one cellulose membrane face were fabricated to make the osmotic pump using conventional protocols as previously described [25]. The adhesion between the PDMS chamber and the cellulose membrane was adhered using the PDMS solution as an adhesive. In preliminary experiments, the osmotic experiments were conducted to evaluate the pumping ability of the osmotic pump. The deionized water was used as a buffer solution and polyethylene glycol (PEG) (Sigma-Aldrich, MO, USA; 2000 molecular weight) solution was used as a driving agent.

### Computational fluid dynamic analysis

The flow distribution during the osmotic pumping was simulated via a computer-aided finite element analysis (FEA), which was constructed using the computational fluid dynamics module in COMSOL Multiphysics 6.0 (COMSOL, MA, USA). For our FEA, two-dimensional (2D) sketches were designed with AutoCAD (Autodesk, CA, USA) layer-by-layer and were subsequently imported to the COMSOL model builder to construct a 3D model. The geometric parameters of this 3D model were shown in Additional file 1: Table S1. The governing equation in the simulation was incompressible Navier–Stokes equations and continuity equation [26]:

1 $$\frac{\partial {\varvec{u}}}{\partial t}+\left({\varvec{u}}\cdot \nabla \right){\varvec{u}}= -\frac{1}{\rho }\nabla P+ \frac{\mu }{\rho }{\nabla }^{2}{\varvec{u}},$$

2 $$\nabla \cdot {\varvec{u}}=0,$$

where **u** is the velocity vector, P is the pressure, and ρ and μ are the density and the dynamic viscosity, respectively. The osmotic pressure was determined by the Van’t Hoff equation [27]:

3 $$\uppi =CRT,$$

where π is the osmotic pressure of the solution, C is the molar concentration of the solute in the solution, R is the molar gas constant [≈ 0.082 (L∙atm)/(K∙mol)], and T is the absolute temperature. Since we employed a PEG solution with a molar concentration of 0.36 M, the osmotic pressure of − 8.654 atm was applied to the outlet in a room temperature.

### Preparation and cell seeding on a gut-liver axis chip

The gut-liver axis chip was sterilized by autoclaving (120 °C for 30 min) and was dried in an oven. The gut chamber of gut-liver axis chip was coated with 1 mg/mL Poly-D-lysine (Sigma Aldrich, MO, USA) for overnight to improve cell adhesion and the liver chamber was coated with 3%(wt/vol) bovine serum albumin (BSA) blocking solution for overnight to improve the uniform-sized hepG2 spheroid generation and inhibit cell adhesion to the bottom PDMS surface. After coating, the gut-liver axis chip was rinsed with a deionized water more than three times, then placed in a dish at 80 °C in an oven at least 24 h. The culture medium was gently and slowly filled into each channel. The human intestinal epithelial cells (Caco-2) (ATCC clone HTB-37) and HepG2 cells were cultured in a modified Eagle’s medium with 10% fetal bovine serum, nonessential amino acids, L-glutamine, and penicillin–streptomycin in the absence of Calcium. Caco-2 cell and HepG2 cell suspension (30 μL, 7.5 × 10^5^ cells/mL) were loaded into the microchannel using a micropipette. The cells in the suspension medium flowed into the microchannel by a gravity and were spontaneously trapped in the microchannel. As the cell suspension with homogenous density was applied in microchannel, the regular amounts of cells were allocated in each microchannel. We left the cells in the incubator for overnight without any treatment for stabilization of cells within the microchannel. After the cells were attached to the microchannel, the non-adherent cells were washed out. The outlet of the gut chamber is connected to the inlet of the filter channel, which is connected back to the inlet of the liver chamber. Each inlet and outlet were connected by a flexible polyurethane tube.

### Immunofluorescence staining

The cells grown in the gut-liver axis chip were fixed with 4% (wt/vol) paraformaldehyde for 30 min, washed twice for 5 min with 0.1% BSA in phosphate buffer saline (PBS), and then permeabilized with 0.2% (vol/vol) Triton X-100 (Sigma Aldrich, MO, USA) for 30 min. After washing with 0.1% BSA in PBS, the cells were incubated with 3% (wt/vol) BSA blocking solution for 1 h. Subsequently, the cells were incubated with primary antibodies overnight at 4 °C, washed three times, incubated with secondary antibodies for 90 min, and washed three times with 0.1% BSA in PBS. The following antibodies were used for immunohistochemistry: rabbit anti-albumin polyclonal antibody (Invitrogen CA, USA, 1:500), Alexa Fluor 594-conjugated phalloidin (Invitrogen, USA, 1:250), and Donkey anti-rabbit Alexa Fluor 488 (Invitrogen, USA, 1:1000). Samples were then incubated with 4′,6-diamidino-2-phenylindole dihydrochloride (DAPI; Molecular Probe, OR, USA) to visualize cell nuclei before taking confocal microscopic images (Olympus, Japan).

### Spheroid viability assay

HepG2 cells are seeded within a microwell array in a gut-liver axis chip and incubated in an incubator for 5 or 10 days to produce uniform-sized 3D spheroids. After the generation of spheroids, the spheroids were stained to measure viability using Live/Dead Cell Assay kit (Sigma-Aldrich, Bayswater, Australia) with staining for 20 min at 37 °C. Calcein-AM can permeate the plasma membrane and is hydrolyzed by the cytoplasmic esterase to form Calcein-AM that can emit the fluorescence. Propidium iodide (PI) is a nucleic acid-staining dye and it cannot permeate the plasma membrane. The stained hepG2 spheroids were imaged under a fluorescence microscope (IX73, Olympus, Japan).

### Functional assessment

Albumin and urea secretion were analyzed by measuring the concentration of albumin and urea in the medium conditioned by culturing with microbiota-derived exosome and hepG2 spheroids. The hepG2 spheroids were cultured within microwell arrays in a fluidic-based gut-liver axis chip. After culturing for 5 and 10 days, the medium collected in the coiled tube to the osmotic pump was analyzed for albumin and urea concentration.

### Bacterial cell culture and live staining

*Lactobacillus paracasei* HY7014 (HY7014) and *Lactobacillus casei* HY7207 (HY7207) probiotics were supplied by hy Co., Ltd. (Yongin-si, Korea). *Lactobacillus paracasei* type strain ATCC25302 (ATCC25302) and *Lactobacillus casei* type strain ATCC393 (ATCC393) from the American Type Culture Collection (ATCC; Manassas, VA, USA) was used a reference strain. *L. paracasei* and *L. casei* strains were grown in Man, Rogosa and Sharp (MRS) broth (BD, Franklin Lakes, NJ, USA) at 37 °C for 24 h. Subsequently, the bacterial cells were harvested by centrifugation (4000 rpm, 10 min, 4 °C), washed three times with PBS, and resuspended in cell culture medium at 10^9^ CFU/mL before each assay. When bacterial cells were harvested by centrifugation (5000 g, 15 min) the cells were resuspended with 2 mL of raw medium. After suspension, the dye mixture of equal volumes (3 μL to each milliliter) of SYTO^®^ 9 and PI (LIVE/DEAD^®^ BacLight™ Bacterial Viability Kit, L7012, Thermo Fisher Scientific) was added. The cells with dye mixtures were incubated at room temperature in the dark for 15 min. After the two-time centrifugation and washing, they loaded to the Caco-2 cell chamber.

### Bacterial-derived EVs purification: Tangential flow filtration (TFF)

*L. paracasei* HY7014 and *L. casei* HY7207 were grown in 1L MRS broth of 37 °C for 24 h and were pelleted by sequential centrifugation at 15,970 g$\times$ at 10 °C for 15 min. The culture supernatants of HY7014 and HY7207 were filtered through a 0.45 μm filter membrane. Bacterial-derived EVs isolation was performed using the KrosFlo^®^ KR2i TFF System from Repligen (Spectrum Labs, Los Angeles, CA, United States) and 500 kDa cutoff TFF filter module (C02-E500-10-N, Spectrum Labs., MicroKros). Briefly, the feed flow rate and transmembrane pressure (TMP) were kept constant at 400 mL/min and 0.5 bar, respectively. The retentate was concentrated into a final volume of 20 mL for 50-fold concentrations.

### Tunable resistive pulse sensing assessment

Quantification and size characterization of EVs were measured using a tunable resistive pulse sensing (TRPS) instrument (Exoid; IZON Science Ltd, Christchurch, New Zealand). Two different nanopores (NP250, NP400, IZON Science Ltd.) were used to assessment in the size range. Carboxylate polystyrene calibration particles (CPC200 and CPC 500, IZON Science Ltd.) were used with NP200 and NP400 nanopores to ensure optimization conditions (e.g., size, concentration). All calibrations and sample measurements were run under the same conditions recommended by the manufacturer and a minimum of 500 particles was recorded at three different pressures. The acquired data was analyzed using Izon Control Suite software (Izon Control Suite version 3.2.2.268, Izon Science Ltd.).

### Preparation and treatment of EVs and Cytotoxicity test

Both microbiota-derived EVs were made up as a 10 × 10^10^ particles/mL stock solution in a culture medium. Then both EVs were diluted in culture medium to give an appropriate final concentration. Briefly, hepG2 spheroids cultured within microwell arrays were treated 0.1, 1, and 10 × 10^9^ particles/mL concentrations until 10 days. After incubation, the cell culture medium transferred into corresponding wells which were optically clear with a 96-well flat bottom plate. LDH activity was measured using a Cytotoxicity Detection Kit (ThermoFisher, MA, USA) according to the manufacturer’s procedure. Finally, the OD at 490 nm with a reference wavelength of 690 nm for each sample was measured. LDH is a soluble cytosolic enzyme that is released into the culture medium following the loss of membrane integrity. LDH activity can be used as an indicator of cell viability. The percentage of LDH release was expressed as the proportion of LDH released into the medium as compared to the total amount of LDH presented in cells that could treat with 2% Triton X-100. It was estimated in the following way:

4 $$Cytotoxicity \left(\%\right)= \frac{Treated \,group - Low \,control}{High \,control - Low \,control} \times 100$$

### Statistical analysis

Data are presented as means ± standard error (SEM). *P*-values were analyzed using Student's t test or one-way ANOVA followed by Tukey's post hoc test (GraphPad Prism version 8.0, GraphPad Software Inc. San Diego, CA, USA). Differences between control groups and experimental groups were considered statistically significant (*p < 0.05, **p < 0.01). Difference between wild type and experimental probiotics were considered statistically significant (^†^*p* < 0.05, ^††^*p* < 0.01). Additionally, difference between microbiota-derived EVs were considered statistically significant (^§^*p* < 0.05).

## Result and discussion

### Development of a gut-liver axis chip and computational fluid dynamic analysis

**Fig. 1:**
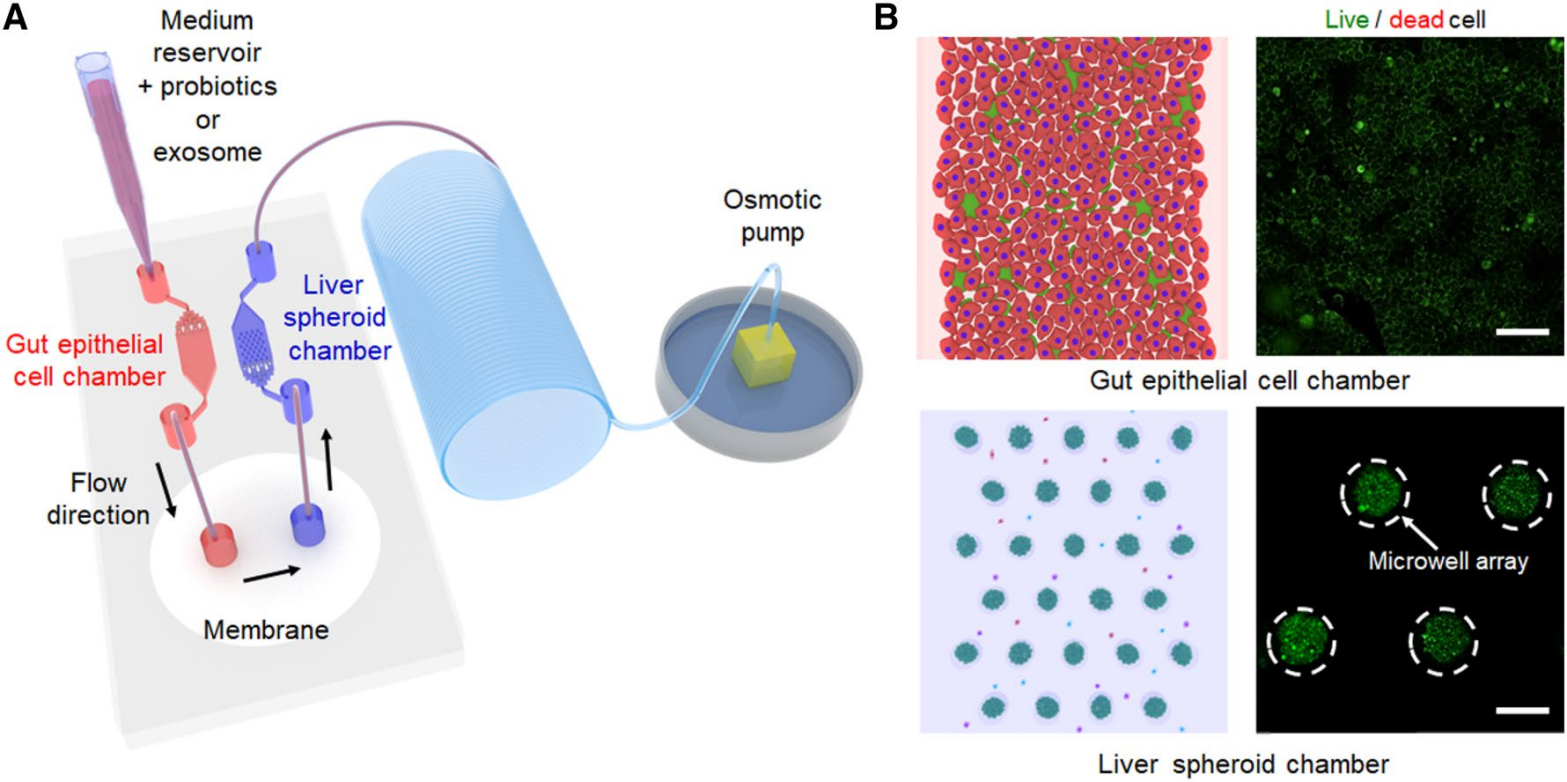
**A** The schematic of experimental setup of a gut-liver axis chip. The left chamber was used to establish an intestinal lumen using human epithelial Caco-2 cells. The right chamber, which contains microwell arrays, was employed to generate the 3D uniform-sized hepG2 spheroids. The continuous flow of the culture medium was introduced by an osmotic pump. **B** Schematic images of gut and liver spheroid chamber as well as representative immunofluorescence results showing that microbial and 3D hepG2 spheroids were located in the left gut chamber and right liver chamber of a gut-liver axis chip separated by cellulose membrane. Scale bar is 100 μm

To investigate the interactions microbiota-derived small molecules on liver in a human intestinal microenvironment, we developed a gut-liver axis chip (Fig. 1A). The gut-liver axis chip consisted of three compartments: intestinal cell chamber, membrane-embedding mass transfer channel, hepG2 spheroid chamber. Intestinal channel (6 mm wide, 20.5 mm long, and 100 μm high) was designed for Caco-2 cell culture and the micropillars were designed for uniform distribution of cells. The intestinal flow is directed to the membrane-incorporated mass transfer channel. Intestinal cells and microbiota are blocked by the cellulose membrane. On the other hand, the culture medium and metabolites pass through the membrane and move to the hepG2 spheroid chamber. In the hepatic spheroid channel (width 6 mm, length 20.5 mm, and height 100 μm), the micropillars were also designed for uniform distribution of cells, and 297 microwell arrays (diameter 200 μm, height 150 μm) were designed to generate uniform-sized hepG2 spheroids. An osmotic pump was used to perfuse the culture medium channel to mimic the fluid flow resulting from interactions of the human intestinal lumen and liver in vivo.

**Fig. 2:**
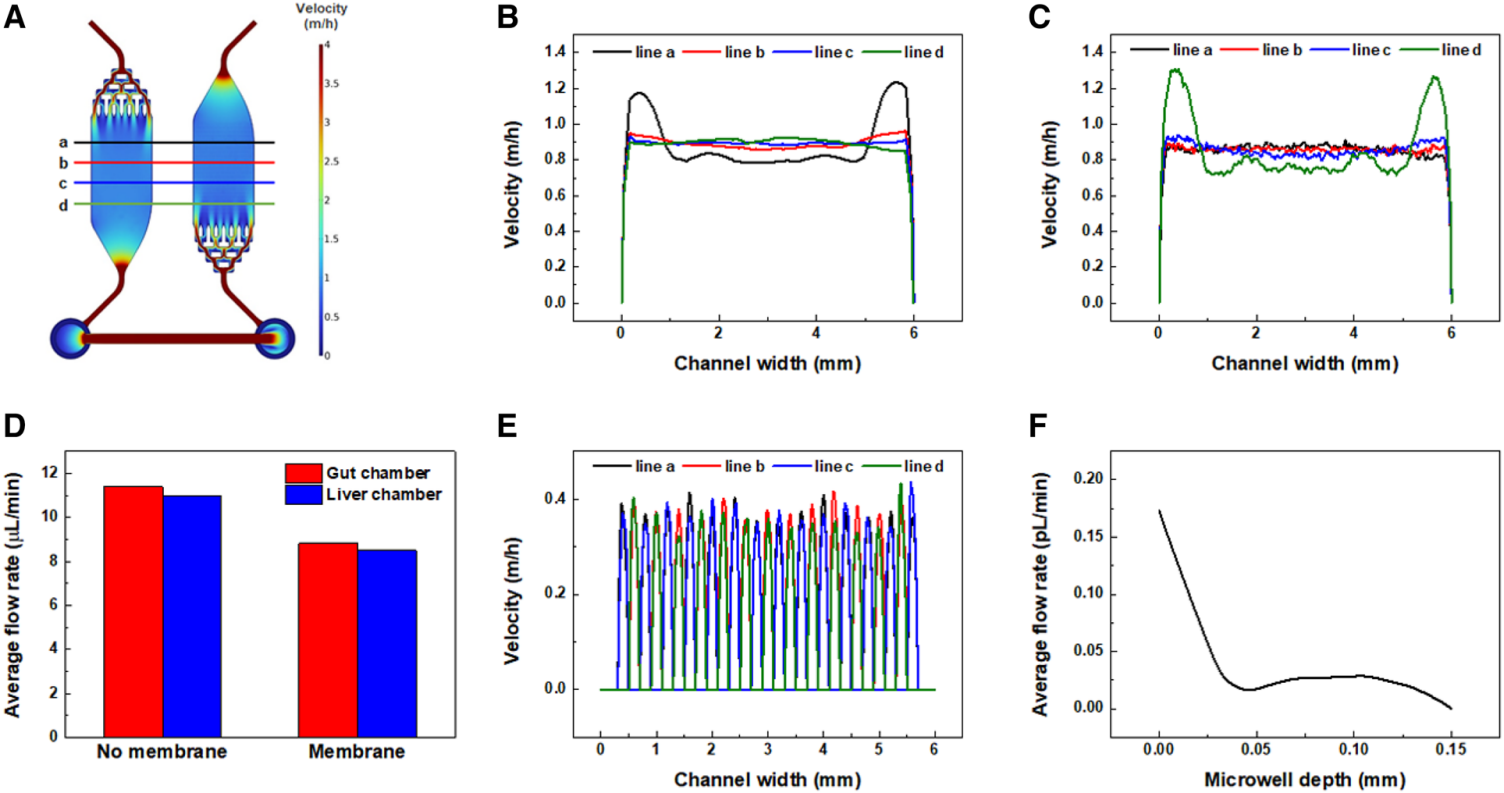
Computational fluid dynamics simulation model for the analysis of flow distribution during osmotic pumping in a gut-liver axis chip system. **A** Flow distribution in a gut-liver axis chip. **B** The velocity profile in the gut chamber. Each dataset was extracted from the respective cutline a, b, c, and d as marked in **A**. **C** The velocity profile in the liver chamber. **D** Comparison graph showing the difference in average flow rate between membrane and no membrane. The insertion of membranes reduced the average flow rate by about 23%. **E** Velocity profile on the top of microwell arrays. **F** Average flow rate profile inside microwell arrays from the top to the bottom of the microwells

We estimated the flow distribution during osmotic pumping via an FEA. Our FEA results highlighted the analogous flow distribution between the gut and liver chambers (Fig. 2A). Velocity profiles at line a, b, c, and d were plotted in the gut (Fig. 2B) and liver chamber (Fig. 2C). In both chambers, the first line where the fluids entered showed different profiles, which could spike up at the edge sides as compared to other lines, because the fluids of those first lines were in an unsteady state. Once the steady state was achieved, the velocity profiles were considerably uniform between the lines. Additionally, it is noteworthy that the velocity profile in our FEA has a flat region where its magnitude is almost unchanged. According to Poiseuille’s law, the velocity profile in the circular tube or parallel plates with infinite walls could be parabolic [28, 29]. However, both chambers of our gut-liver axis chip were rectangular-shaped geometry with a large aspect ratio (600:1). A theoretical solution of the velocity profile in a rectangular cross-section (w > h) could be derived via the expansion of the Navier–Stokes equation with Fourier series [30]:

5 $${v}_{x}\left(y, z\right)= \frac{4{h}^{2}\Delta P}{{\pi }^{3}\mu L}{\sum }_{n, odd}^{\infty }\frac{1}{{n}^{3}}\left[1- \frac{\mathrm{cosh}\left(n\pi \frac{y}{h}\right)}{\mathrm{cosh}\left(n\pi \frac{w}{h}\right)}\right]\mathrm{sin}\left(n\pi \frac{z}{h}\right),$$

where L, h, and w refer to the length, height, and width of the rectangular channel, respectively. Edge effects by finite walls hindered the parabolic development of the velocity profile; hence it was elucidated that the velocity profile in a rectangular channel showed a flat region at the center in the long width while being parabolic in the short height. Since most microchannels had been fabricated with a large aspect ratio, our gut-liver axis chip could confer an advantage in providing uniform quantities of fluid regardless of the locations. We also observed that the flow rates inside both culture chambers were decreased by about 23% when intercalating membranes between the fluidic layers (Fig. 2D). Since the osmotic pump generates the pressure-driven flow, the increased channel resistance by membrane insertion can lead to a compromise in the flow rate. Interestingly, the properties of membranes, such as pore size or porosity, did not affect the flow rate (Additional file 1: Fig. S1A, B), showing that the types of membranes were not important in an aspect of flow distribution. Moreover, lower flow rates in the liver chamber than in the gut chamber showed that some of the fluids flowed into the microwell arrays. It was demonstrated by the velocity profiles on the top of the microwell arrays (Fig. 2E) and the average flow rate profile across the depth of microwell arrays (Fig. 2F). In particular, the analogous slip velocities among the tops of the microwell arrays showed that almost uniform fluids entered the respective microwells.

### Effect of physiological flow rate on probiotics

**Fig. 3:**
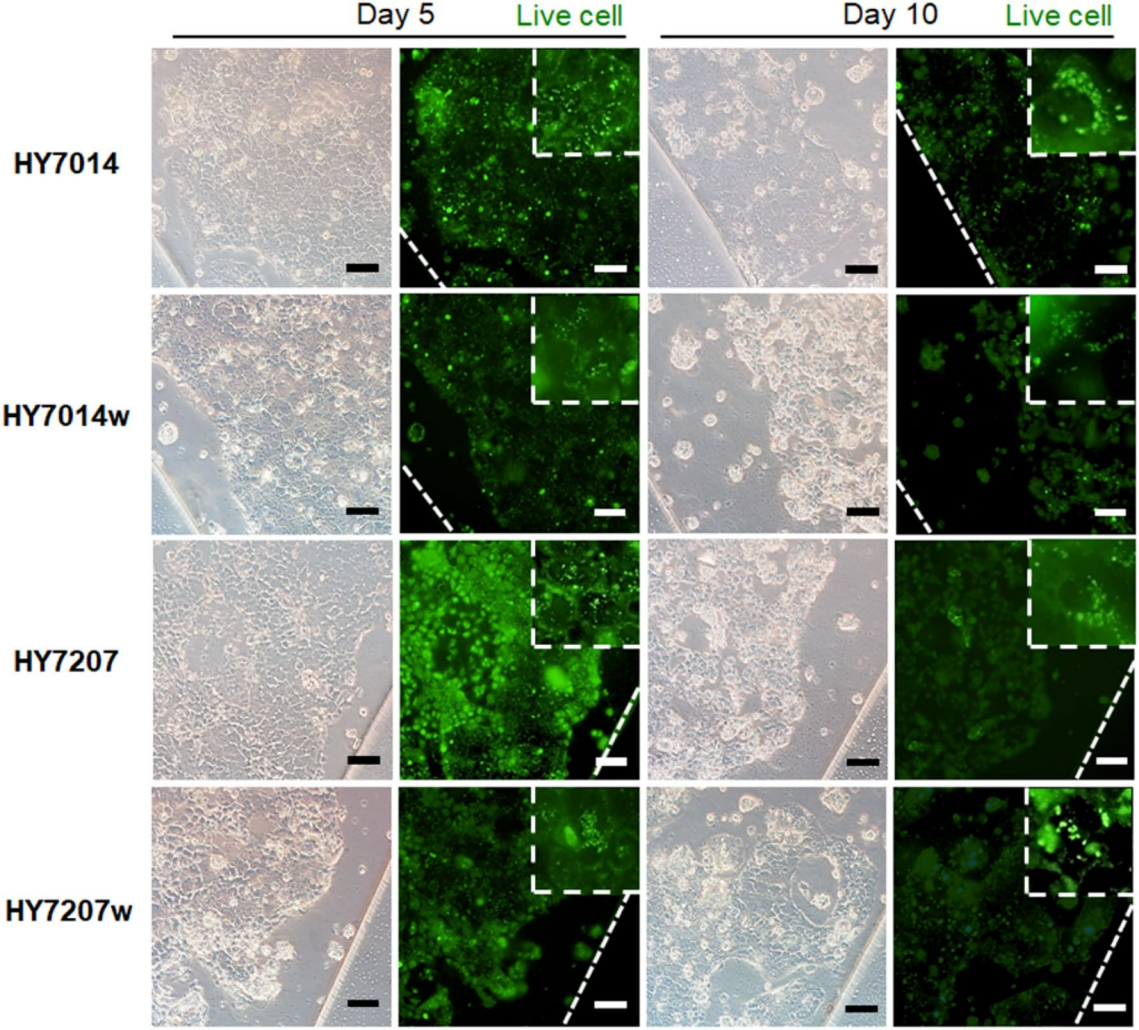
Microbial co-culture on a human intestinal epithelial layer in a gut-liver axis chip. Both wild type and experimental microbial were cultured on the surface of an epithelial layer grown within a gut-liver axis chip (HY7014 and HY7207 probiotic; HY7014w and HY7207w as a control of the wild type; seeding density 1 × 10^9^ CFU/mL). Green fluorescence views from above of probiotics and Caco-2 cell co-cultured for 5 and 10 days at low and high (white-dotted rectangle) magnification, which shows microcolonies of the microbial (green spots) that remain tightly adherent to the apical surface of the epithelial layer after exposure to continuous fluidic flow. Scale bars are 100 μm

To determine the stability of the gut-liver axis chip system for the long-term culture, we explored the adhesion and colonization of the commensal microbiota in a flow culture condition. Bacterial overgrowth occurs rapidly (within ~ 1 day) and compromising the epithelium layer [31, 32]. Thus, there has been needed to achieve long-term cultures and avoid bacterial overgrowth for more robust recapitulation of microbiota-host cell interactions. We employed a gut-liver axis chip that enables Caco-2 cells and microbiota to be cultured in the presence of physiologically relevant luminal flow. In this study, *L. paracase* HY7014 probiotic and *L. casei* HY7207 probiotic were employed and we also used the wild types of each strain as a control. Blocking the fluidic flow for the first 2 h, the bacterial cells were allowed to adhere on the apical surface of the villi. After 2 h, the physiological relevant flow was resumed through the microchannels to remove un-colonized probiotics. When a laboratory strain of the green fluorescent stained both probiotics was allowed to adhere to the apical surface of the villi for 2 h under static conditions, the bacteria cells were subsequently colonized and spontaneously formed in inhabited regions (Fig. 3). When the probiotics were cultured on the villus epithelium layer under the previously optimized flow condition (21 μL/h) as previously reported in our research group [33], we observed the colonized stable form until day 5 in all probiotic groups. However, both wild type probiotics seemed detached from the epithelial layer and washed out after 10 days when cultured under flow conditions as compared to experimental probiotics, although the luminal flow was maintained constant. All species showed the adhesion to the epithelial layer. However, the adhesion level of the experimental probiotic was greater to epithelial layer even than wild type of probiotics in day 10. These results demonstrated that fluidic flow in our gut-liver axis chip enabled the continuous culturing of epithelial cells and colonized microbiota, while avoiding bacteria overgrowth.

### Morphological observation and viability of HepG2 spheroids cultured in flow culture condition

**Fig. 4:**
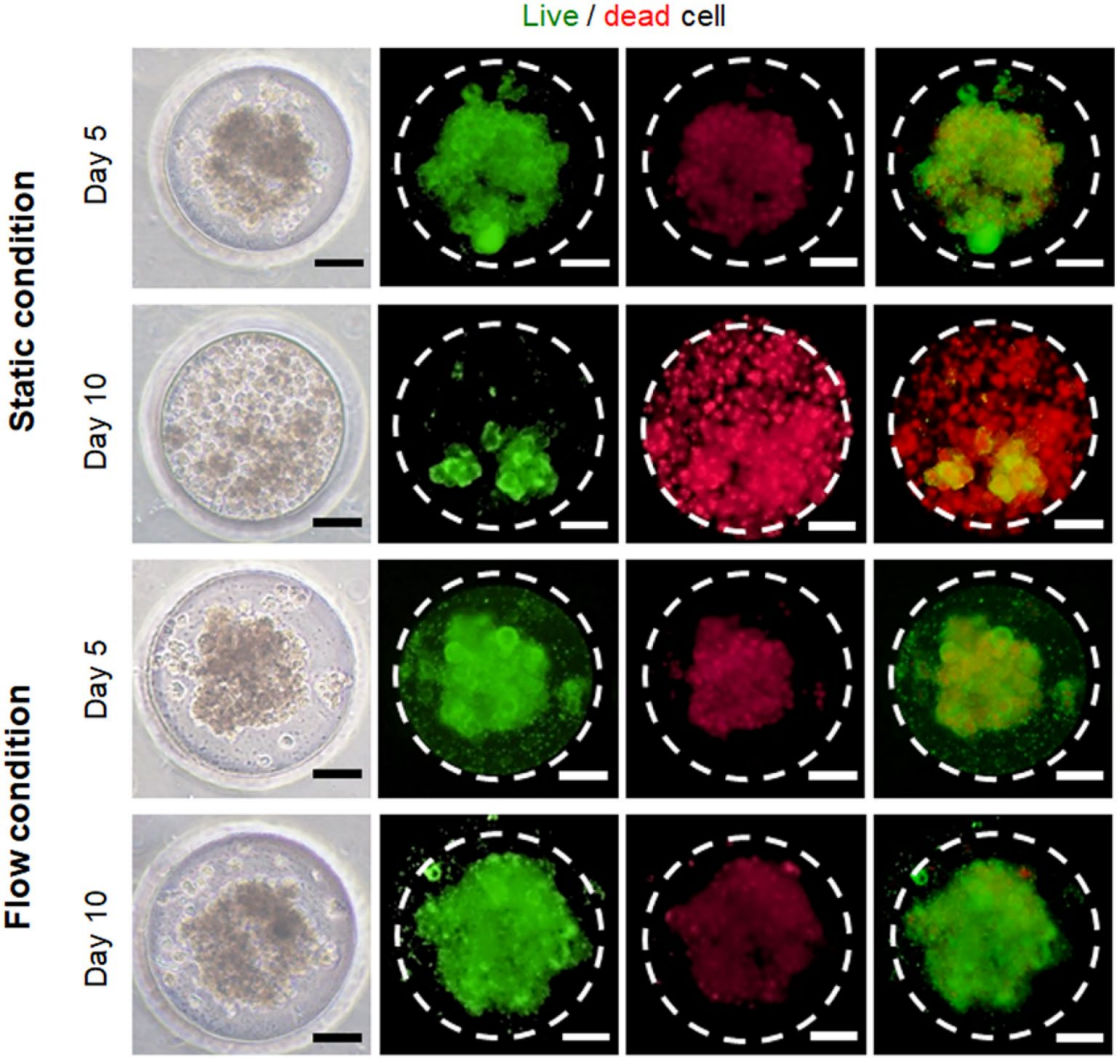
HepG2 spheroid formation within microwell arrays after 5 and 10 days in a gut-liver axis chip. Light microscopic images of hepG2 spheroids formed in microwell arrays. Spheroid image after cultured in flow and static condition (live and dead cells were stained with green and red, respectively). Scale bars are 50 μm

We investigated the biological feature of uniform-sized HepG2 spheroids within microwell array in a gut-liver axis chip. The microwell arrays were uniformly filled with hepG2 cells in a suspension, the cells were subsequently cultured overnight in an incubator without any flow perfusion to allow for uniform-sized hepG2 spheroids formed within microwell arrays. The hepG2 cells cultured in the microwell arrays were physically constrained and were spontaneously formed spheroids in a homogeneous size manner, which was consistent with other previous studies [34, 35]. As shown in Fig. 4, the optical images showed that the fluidic flow allowed for hepG2 spheroids with smooth surfaces and maintained their morphological feature after 10 days. A live/dead cell viability assay was performed to confirm the viability of the spheroid in a fluidic culture condition at 5 and 10 days. (Fig. 4) The viability of the hepG2 spheroids cultured in a flow culture condition after culturing for 5 and 10 days was approximately 100% and 91%, respectively (Additional file 1: Fig. S2). Additionally, the viability of hepG2 spheroids exposed to the fluidic flow condition was higher than that of the spheroids cultured in a static condition (94% and 24% in static culture condition, respectively), suggesting that the fluidic flow could enhance the viability of a uniform-sized 3D hepG2 spheroid. Furthermore, hepG2 spheroids cultured in a static condition did not maintain the spheroidal shape after 10 days of culture. This result clearly demonstrated that this gut-liver axis chip could minimize the spheroid loss and maintain the cell morphology and viability in a long-term perfusion culture.

### Effect of metabolized medium through gut-microbiota compartment on liver spheroid functions

**Fig. 5:**
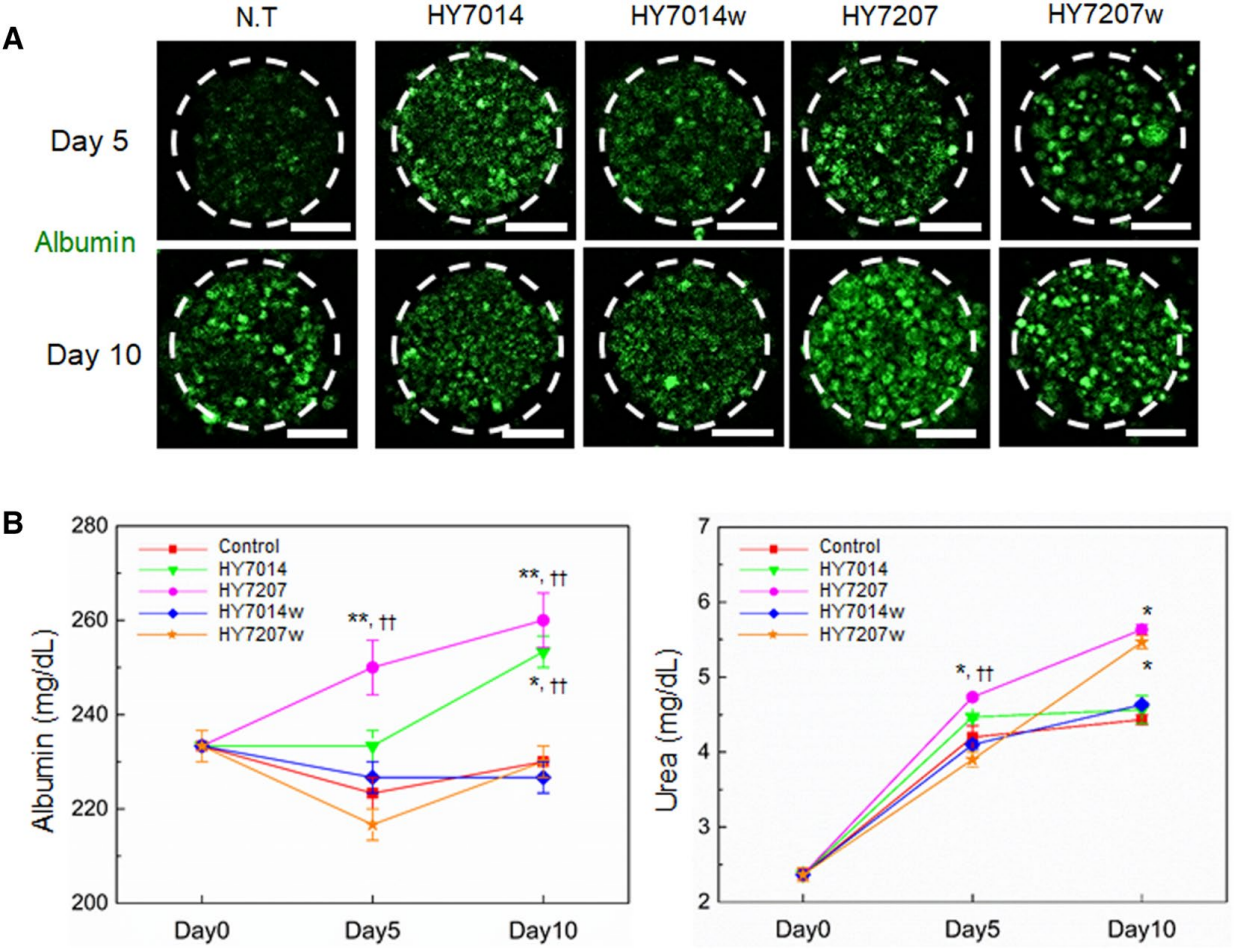
**A** Immunostaining for serum albumin (green) in the microbial co-cultured spheroids for 10 days. The nuclei were stained with DAPI (blue). Scale bars are 50 μm. **B** Analysis of the function of microbial co-cultured spheroids, measuring in terms of the secretion of albumin (left) and urea (right). Data are represented as the mean ± standard error (SEM) of three independent experiments. *p < 0.05 for control *vs.* each group, **p < 0.01 for control *vs.* each group, and ^††^*p* < 0.01 for wild type *vs.* each experimental microbiota

Gut microbiome and gut-derived metabolites play crucial roles in maintaining the physiological functions of host. The beneficial or harmful effect of specific microbiota-derived metabolites depends on the context and the host state [36]. To determine the relationship between hepG2 spheroids and gut microbiota-derived metabolites, we established five experimental groups: (1) control (N.T), (2) HY7014, (3) HY7014 wild type (HY7014w), (4) HY7207, and (5) HY7207 wild type (HY7207w). Gut-microbiota co-culture compartment and hepG2 spheroid culture compartment separated by a porous membrane, which makes it possible to be affected only by metabolized substances. We compared the performance of hepG2 spheroids cultured with various types of gut-microbiota-derived metabolites supplemented medium by measuring secreted albumin and urea concentrations, showing the significant microbiota type-dependent differences. Of the five models, the control and both wild type group lost their hepatocyte-specific functions most rapidly in 5 days. Albumin secretion of hepG2 spheroids were restored to some extent on the 10 days, but it was not significant. In contrast, HY7014 and HY7207 group improved their function for a longer period than control group and wild type groups. The liver spheroids in both HY7014 and HY7207 experimental probiotics group exhibited a greater degree of albumin production than the control spheroids (Fig. 5A) after 10 days of culture. Furthermore, the HY7207 group exhibited the highest albumin and urea production after 10 days (**p < 0.01, *p < 0.05, respectively) (Fig. 5B). When the experimental group and the wild type group were compared, the secretion of albumin and urea was significantly increased in the experimental group. As a result, we demonstrated that HY7207 group showed excellent liver functions as compared to other groups. Recently, the probiotic secondary metabolites, known as postbiotics, have gained great interest due to their potential beneficial materials in humans like the prevention of disease [37]. Thus, HY7207 probiotics could inhibit the hepatocyte apoptosis and accumulation of lipids in hepatocytes, which could protect the hepatocyte cells [38]. Therefore, these results demonstrated that metabolites driven from different types of microbiota could affect the preservation of hepatocyte function via various mechanism.

### Characterization of gut microbiota-derived EVs

**Fig. 6:**
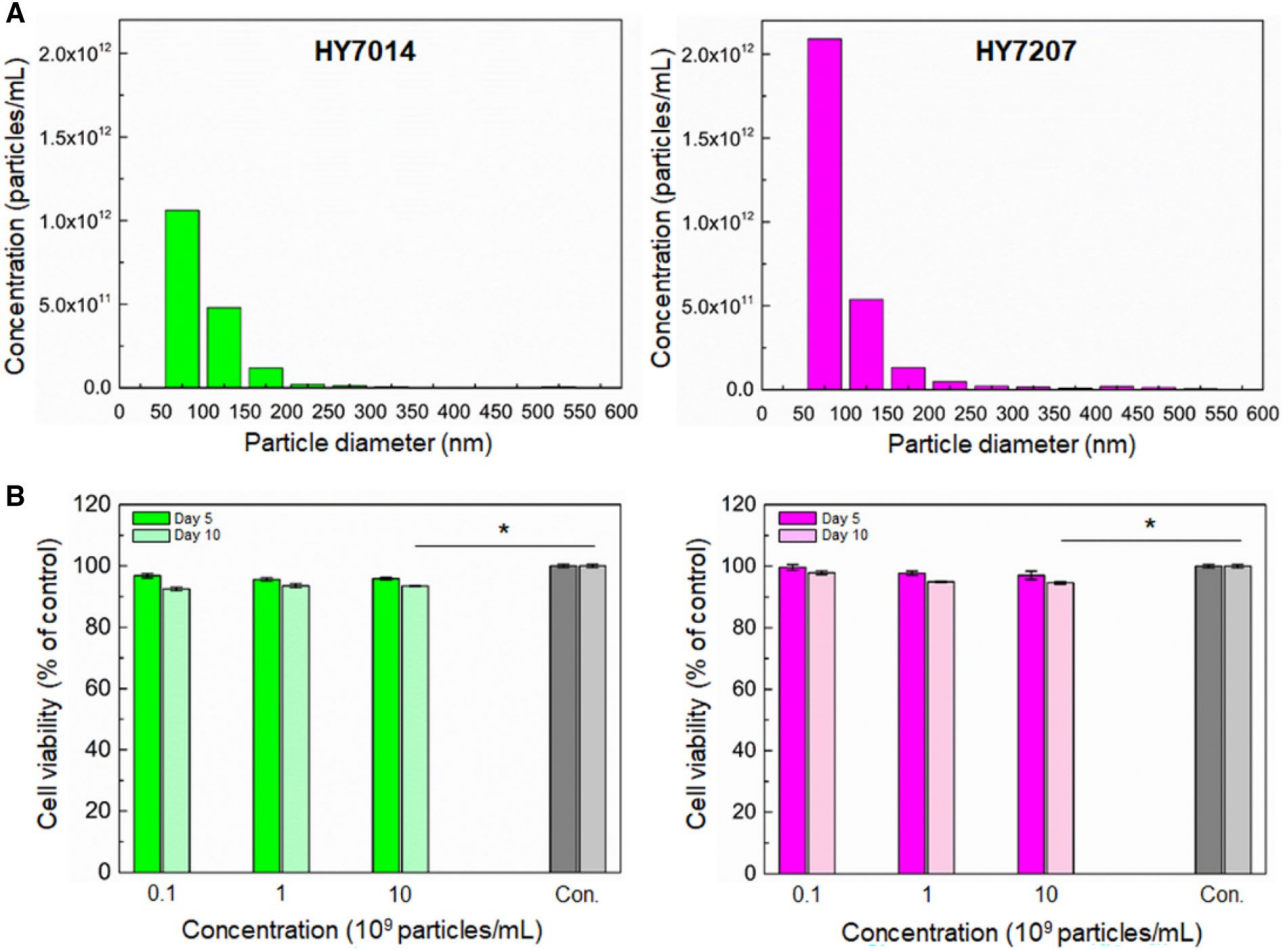
Characterization of microbiota-derived extracellular vesicles. **A** Size distribution analysis of exosome-enriched EV samples analyzed by TRPS system. **B** Percentage of viable cells, measured as LDH release, after treatment with various concentrations of EVs (0.1 × 10^9^ to 10 × 10^9^ particles/mL), Data are represented as the mean ± SEM of four independent experiments. *p < 0.05 for control *vs.* each group

We harvested the supernatant of microbiota and isolated EVs by a TFF method. We evaluated the actual numbers and sizes of particles which could be derived from the culture supernatant of HY7014 and HY7207 using TRPS system in which samples were driven through the 250 nm sized nanoporous membrane (NP250) by applying the different pressures. We detected the particles ranging in size between 50 and 600 nm in HY7014 and 50 nm and 550 nm in HY7207. HY7014 produced at least 2 × 10^11^ particles/mL of EVs consisting of a cytoplasmic membrane with a size range of 50 ~ 600 nm in a culture medium. HY7207 produced at least 5 × 10^11^ particles/mL of EVs consisting of a cytoplasmic membrane with a size range of 50 ~ 550 nm in a culture medium (Fig. 6A). *Lactobacillus acidophilus* ATCC 53544, *L. casei* ATCC 393, and *L. reuteri* ATCC 23272 were previously reported to produce between 3 × 10^9^ and 1 × 10^10^ particles/mL of EVs in each culture medium [39]. The concentration of both microbiota EVs detected in a culture medium was higher than those of EVs from these lactobacilli in a culture medium. Additionally, we analyzed the size distribution of exosomes, showing the mean vesicle diameters of 100.7 nm and 97.3 nm in HY7014 and HY7207, respectively. Furthermore, the mode diameters of each microbiota-derived EVs were revealed 79.7 nm and 71.3 nm, respectively (Table 1). To eukaryotic EVs, bacterial-derived EV size range exclusively below 300 nm in diameter, as previously described [40]. We confirmed that the size of EVs derived from bacterial cells provided from HY company in Korea was corresponded to the previous result.

**Table 1:** Sze distribution analysis of EVs

Contents	HY7014 Exo	HY7207 Exo
Mean peak diameter	100.7 nm	97.3 nm
Mode peak diameter	79.7 nm	71.3 nm
Concentration	1.7 × 10^12^ particles/mL	2.8 × 10^12^ particles/mL

### Effect of gut microbiota-derived EVs on function of hepG2 spheroids

**Fig. 7:**
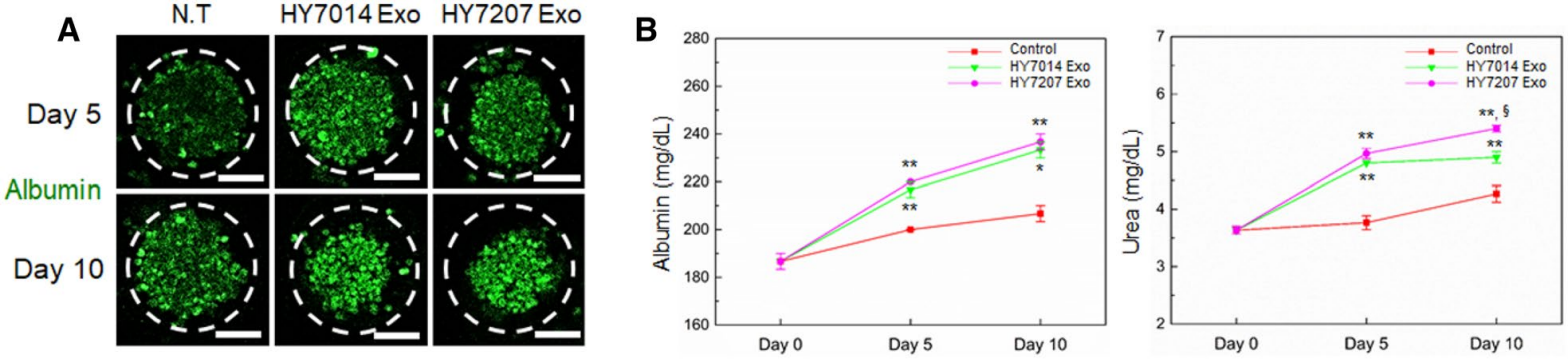
**A** Immunostaining for serum albumin (green) in the hepG2 spheroids treated with various microbiota-derived EVs for 10 days. The nuclei were stained with DAPI (blue). Scale bar are 50 μm. **B** Analysis of the function of microbial co-cultured spheroids measuring in terms of the secretion of albumin (left) and urea (right). Data are represented as the mean ± SEM of three independent experiments. *p < 0.05 for control *vs.* each group, **p < 0.01 for control *vs.* each group, and ^§^*p* < 0.05 for HY7014 Exo *vs.* HY7207 Exo

In present, there is still limited understanding of the toxicity and immunogenic potential of EVs [41, 42]. Thus, it needs in-depth understanding of their safety and toxicity profile. To determine the potential biological properties of microbiota-derived EVs to influence hepatocyte viability, HepG2 cells were treated with various concentrations of EVs (0.1 × 10^9^ to 10 × 10^9^ particles/mL) to analyze the cell viability and liver functions. HepG2 cell viability assessed by released LDH after 5 and 10 days culturing with EVs remained similar among different EV doses (Fig. 6B). Indeed, EV treatment did not have a detrimental effect on HepG2 cell viability. However, a modest toxicity effect was observed at the highest EV concentration (10 × 10^9^ particles/mL) at 10 days as compared to the control. The functional activity of HepG2 cells was then assessed by immunostaining and quantification of albumin and urea secretion after different types of microbiota-derived EVs treatment. The EVs-treated hepG2 spheroids exhibited a greater degree of albumin production than the EVs-free (control) spheroids (Fig. 7A) after 10 days of culture. This result was confirmed by a quantitative analysis of the albumin and urea secretion, showing greater secretion of albumin in the EVs-treated hepG2 spheroids than in the EVs-free (control) spheroids (Fig. 7B). Additionally, the albumin secretion in all EVs-treated spheroids was significantly improved in 10 days after culturing than day 5 (**p < *0.01*), while there was not significantly different secretion of the albumin between treatment with different types of EVs. Furthermore, in urea concentrations on day 5 and 10, the hepG2 spheroids treated with microbiota-derived EVs exhibited significantly higher values (up to 5.1 mg/dL in day 5, up to 5.5 mg/dL in day 10) of urea synthesis as compared to EVs-free spheroids. Moreover, the concentration of urea in hepG2 spheroids treated with HY7207-derived EVs was significantly higher than spheroids treated with HY7014-derived EVs (*p < *0.05*). The EVs-treated hepG2 spheroids showing a higher value for urea synthesis indicated that a greater functionality as compared to the EVs-free spheroids [43]. Microbiota-derived EVs are known to useful transporter to transfer biological active compounds between the cells [44–46]. It has been known that the exosome, an endosomal-derived small membrane vesicle, have an important role as a mediator in cell–cell communications, affecting a large number of pathways in the recipient cells [47, 48]. However, through their biogenesis, EVs can contain various cell-derived bioactive cargoes that have the potential to cause undesirable effects in recipient cells, such as toxicity and immune-stimulation [49, 50]. Our result clearly demonstrated that hepG2 spheroid function was not impaired in the presence of EVs, regardless of the EV concentrations, with no signs of cytotoxicity after 10 days treatment. These results suggested that microbiota-derived EVs might be positively involved in the cellular behavior or response, such as viability and functions.

## Conclusions

We demonstrated that the cell viability and liver functions were significantly enhanced in response to microbiota-derived small molecule treatment in our gut-liver axis chip. We isolated microbiota EVs and observed their positive effect on hepG2 cell survival and maintenance of liver functions. We also observed that these microbiota-derived small molecules could alter the albumin and urea secretion within recipient cells. Therefore, our gut-liver axis chip could provide a helpful and efficient co-culture platform to study the interactions of microbiota-derived small molecules and recipient cells.

## Supplementary Information

**Additional file 1.**Table S1. Geometric parameters of the 3D computational fluid dynamics model. Figure S1.Comparison graph according to (A) pore size and (B) porosity of membranes. Figure S2. Effects of the fluidic flow onviability in hepG2 spheroids.

## Data Availability

The authors have no data to share since all data are shown in the submitted manuscript.

## Abbreviations

GI: Gastrointestinal
EVs: Extracellular vesicles
SCFAs: Short-chain fatty acids
Caco-2: Intestinal epithelial cell
TFF: Tangential flow filtration
TRPS: Tunable resistive pulse sensing
LDH: Lactate dehydrogenase
