# Supplementary material for: Effect of gut microbiome-derived metabolites and extracellular vesicles on hepatocyte functions in a gut-liver axis chip
Source: Nano Converg. 2023 Jan 16;10:5. doi: 10.1186/s40580-022-00350-6 (PMC9842828; doi:10.1186/s40580-022-00350-6)
Supplement: Supplementary file 1 — Additional file 1.Table S1. Geometric parameters of the 3D computational fluid dynamics model. Figure S1.Comparison graph according to (A) pore size and (B) porosity of membranes. Figure S2. Effects of the fluidic flow onviability in hepG2 spheroids. [file 40580_2022_350_MOESM1_ESM.docx]

**Supporting information**

| Contents | Dimension |
| --- | --- |
| Porosity of membrane [%] | 50 |
| Pore size of membrane [μm] | 0.22 |
| Thickness of membrane [μm] | 60 |
| Width×height of culture chamber [mm×mm] | 6×0.1 |
| Radius×depth of microwell array [mm×mm] | 0.1×0.15 |

**Table S1**. Geometric parameters of the 3D computational fluid dynamics model.

**
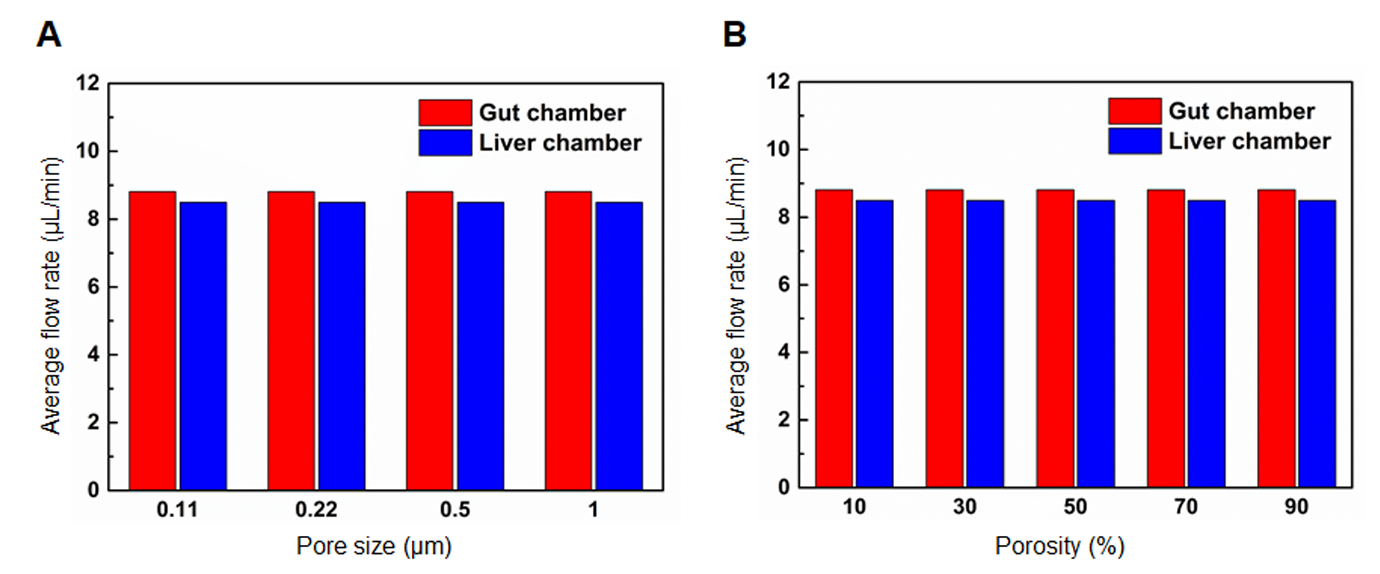
**

**Figure S1.** Comparison graph according to (A) pore size and (B) porosity of membranes.


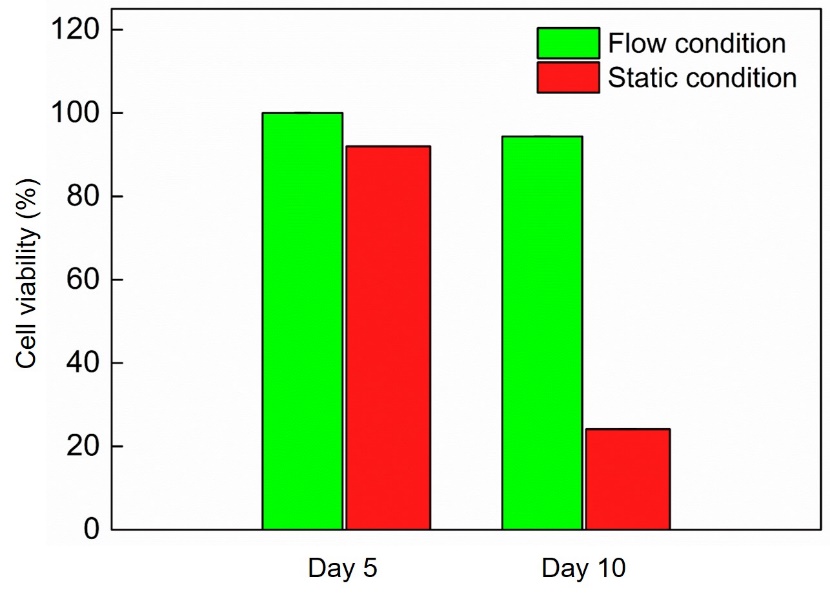


**Figure S2.** Effects of the fluidic flow on viability in hepG2 spheroids.
